# Recovered Patients with Stevens–Johson Syndrome and Toxic Epidermal Necrolysis Maintain Long-Lived IFN-γ and sFasL Memory Response

**DOI:** 10.1371/journal.pone.0045516

**Published:** 2012-09-18

**Authors:** Meng Fu, Yang Gao, Yuefei Pan, Wei Li, Wenjun Liao, Gang Wang, Chunying Li, Chengxin Li, Tianwen Gao, Yufeng Liu

**Affiliations:** 1 Department of Dermatology, Xijing Hospital, The Fourth Military Medical University, Xi'an, People's Republic of China; 2 Department of Health Science, School of Military Preventive Medicine, The Fourth Military Medical University, Xi'an, People's Republic of China; Institut Jacques Monod, France

## Abstract

There is evidence that drug-specific T cells are involved in inducing keratinocyte apoptosis in acute stage of Steven-Johson syndrome (SJS) and Toxic epidermal necrolysis (TEN). However, there are few studies that have attempted to examine T cell memory responses over time. We sought to determine the duration of IFN-γ and sFasL memory response to causal drugs in patients with SJS and TEN after remission. Eight patients with previous SJS and TEN were enrolled. Memory T cells were measured by 10-day cultured IFN-γ enzyme-linked immunosorbent spot-forming cell (ELISpot) assay. Effector T-cell responses were measured by *ex vivo* IFN-γ ELISpot assay and sFasL ELISA. The sFasL-mediated toxicities of drug-stimulated PBMC supernatants against keratinocyte line were further investigated by MTT proliferation assay and Annexin-V staining. We observed significant cultured and *ex vivo* IFN-γ ELISpot responses against causal drugs in all 8 patients. In addition, the sFasL levels were specifically increased in the supernatant of PBMCs cultured with causal drugs from 6 of 8 patients. Drug-stimulated PBMC supernatants were cytotoxic against keratinocyte line, which was inhibited by anti-FasL mAb in a dose-dependent manner. Our findings confirmed that drug-specific IFN-γ and sFasL memory response against causal drugs could be sustained over several years and further suggest that patients should avoid causal drug re-exposure after the recovery of TEN and SJS.

## Introduction

Stevens-Johnson syndrome (SJS) and toxic epidermal necrolysis (TEN) are the most severe forms of drug induced skin diseases and are now considered variants of the same disease. The most common feature of these diseases is the formation of subepidermal blisters and detachment of the epidermis, which appears as scalded skin. Necrosis of the full thickness of the epidermis is the pathognomonic finding in this entity. The death rates average 25%, and many survivors have sequelae.

It has been well established that the epidermal damage in these diseases is due to keratinocyte apoptosis. The mechanism is not fully understood, but it is believed to be mediated by drug-specific T cells. Drugs stimulate T cells either by forming hapten-protein complexes presented by MHC or possibly by direct binding to T cell receptors [1], [2]. T cells utilize granulysin/perforin/granzyme B trigger keratinocyte apoptosis [3], [4], [5], [6]. Moreover, T cells express death-inducing protein Fas ligand (FasL), which induces apoptosis by engaging Fas (CD-95) on keratinocytes [7], [8]. In addition, T cell is able to activate keratinocyte by producing IFN-γ, TNF-α, which render keratinocytes susceptible to apoptosis induced by perforin/granzyme B and sFasL [5], [9].

Although the contribution of T cells to SJS and TEN has been extensively studied, the development and maintenance of drug-specific memory response in SJS and TEN patients remains to be elucidated. Using the lymphocyte toxicity assay (LTA), Halevy et al has revealed that the patients with ibuprofen-induced SJS or sulfonamides-induced hypersensitivity syndrome present a high level of cytotoxicity to the incriminating drugs over several years after remission. Furthermore, the incriminating drugs can markedly stimulate the PBMCs to produce a variety of pro-inflammatory cytokines [10], [11]. It strongly points to the maintenance of effector memory response to the causal drugs in patients with cutaneous drug adverse reactions (CADRs). Beeler et al reported that drug reactivity in patients with severe systemic drug hypersensitivity persists for a long period, and the frequency of drug-reactive T cells is about 1∶250 to 1∶10,000 of T cells [12]. However, only one SJS patient was included in the study. Thus, it makes it difficult to determine the longevity and frequency of drug-specific memory T cells for SJS and TEN. Zawodniak A et al demonstrated that granzyme B could be released by PBMCs from patients with various drug-induced skin diseases in remission [13]. The granzyme B response tend to be low, and IL-15 is needed to enhance granzyme B release. Whether the results reflect a decreased memory response remains to be elucidated.

Understanding the persistence and effector function of memory T cells is fundamental for *in vitro* diagnosis of SJS and TEN. In this study, we selected 8 patients with a very well characterized medical history of SJS and TEN and with positive lymphocyte transformation test (LTT) to the causal drugs. The memory T cells was exploited by cultured IFN-γ enzyme-linked immunosorbent spot-forming cell (ELISpot) assay [14], [15]. The effector function was exploited by *ex vivo* overnight IFN-γ ELISpot assay [16] and by sFasL ELISA [8]. We also incubated the supernatants of drug-stimulated PBMCs with HaCat cells and anti-FasL blocking mAb to determine sFasL-mediated toxicity against keratinocytes. We sought to determine whether IFN-γand sFasL memory response could be maintained in SJS and TEN patients after remission.

## Results

### Patient characteristics

We recruited 8 patients with a very well characterized medical history of SJS and TEN. Table 1 details the age, gender, culprit drugs, disease and intervals between acute allergy and present analysis. All 8 patients were analyzed in clinical remission of SJS and TEN and were healthy at the time of analysis. The interval between the occurrence of SJS and TEN and the present analysis varied from 1 month to 3 years. Six patients who had a history of drug-induced maculopapular exanthema (MPE) were also included in the study. The interval between the acute allergy and the present analysis ranged from 1 month to 2 years (Table 1).

**Table 1:** Patient characteristics.

Patient	Age/ gender	Causal drugs	Irrelevant drug	Disease	LTT	Intervals^*^
1	32 years/f	CFZ (50 ug/ml)	NMS (7 ug/ml)	TEN	+	3 years
2	37 years/f	NMS (7 ug/ml)	CFZ (50 ug/ml)	TEN	+	2 years
3	57 years/f	AMX (40 ug/ml)	NMS (7 ug/ml)	SJS	+	1 year
4	80 years/m	PNC (40 ug/ml)	NMS (7 ug/ml)	TEN	+	1 year
5	64 years/m	NMS (7 ug/ml)	CFZ (50 ug/ml)	SJS	+	1 year
5	3 years/m	APAP (50 ug/ml)	CFZ (50 ug/ml)	TEN	+	1 year
6	77 years/m	AP (50 ug/ml)	CFZ (50 ug/ml)	TEN	+	3 month
7	26 years/m	AMX (40 ug/ml)	NMS (7 ug/ml)	SJS	+	2 months
8	15 years/f	HCQ (40 ug/ml)	CFZ (50 ug/ml)	TEN	+	1 month
9	48 years/f	CBZ (25 μg/mL)	AMX (40 ug/ml)	MPE	+	2 years
10	46 years/m	APAP (50 ug/ml)	CFZ (50 ug/ml)	MPE	+	1.5 years
11	21 years/f	AMX (40 ug/ml)	NMS (7 ug/ml)	MPE	+	1 year
12	45 years/m	PNC (40 ug/ml)	NMS (7 ug/ml)	MPE	+	1 year
13	34 years/f	CBZ (25 μg/mL)	AMX (40 ug/ml)	MPE	+	3 months
14	14 years/f	CFZ (50 ug/ml)	NMS (7 ug/ml)	MPE	+	1 month

Drug concentrations used for stimulation in *in vitro* experiments. f, female; m, male; CFZ, cephazolin; NMS, nimesulide; AMX, amoxicillin; PNC, penicillin; APAP, acetaminophen; HCQ, hydroxychloroquine; CBZ, carbamazepine; AP, allopurinol; TEN, toxic epidermal necrolysis; SJS, Stevens–Johnson syndrome; MPE, maculopapular exanthema; LTT, lymphocyte transformation test. *Interval between acute allergy and present analysis.

### IFN-γ production by drug-specific memory T cells

**Figure 1:**
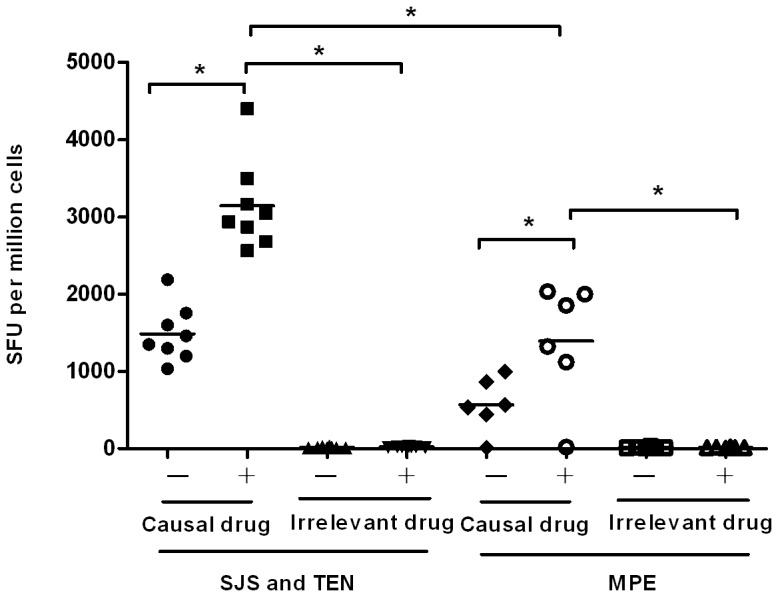
Cultured IFN-γ ELISpot responses to causal drugs. PBMCs from patients with SJS and TEN or MPE were incubated with either causal drugs or irrelevant drugs for 10 days. The cells were subsequently washed, and then stimulated with causal drugs or irrelevant drugs in an IFN-γ ELISpot assay. Responses are presented as number of IFN-γ-positive spot-forming units (SFU) per million cells. Each data point represents an individual patient with SJS and TEN or MPE. Median is indicated by a horizontal line. *P<0.01.

To investigate the durable memory of drug-specific T cells, PBMCs from eight SJS and TEN patients in remission were cultured with causal drugs for 10 days in vitro. At the completion of the culture period, the cultured T cells were restimulated for 24 hr in the presence of the causal drugs. Responding T cells were then examined by ELISpot for the production of IFN-γ. In all 8 patients with SJS and TEN, the cultured T cells were activated to produce IFN-γ in the restimulation phase of the assay. The response ranged from 2565 to 4400 SFU per million T cells, as shown in Fig. 1. Interestingly, even if without restimulation with causal drugs, the cultured T cells still showed a capacity of producing IFN-γ, although we washed cells sufficiently well and left them overnight. In contrast, the T cells cultured with chemically irrelevant drugs failed to elicit significant IFN-γ response. There was no significant correlation between cultured IFN-γ ELISpot response and the time interval from previous SJS and TEN. A substantial cultured IFN-γ ELISpot response was observed as long as 3 years later in patient 1 who had strict drug avoidance. In patients with previous MPE, the cultured IFN-γ ELISpot responses were detected in 5 of 6 subjects (Fig. 1). The positive responses ranged from 1123 to 2034 SFU per million T cells, which were significant lower than those in SJS and TEN group. Overall, these data suggest that drug-specific T cell memory responses are stably maintained in SJS and TEN patients after remission.

### IFN-γ production by drug-specific effector memory T cells

**Figure 2:**
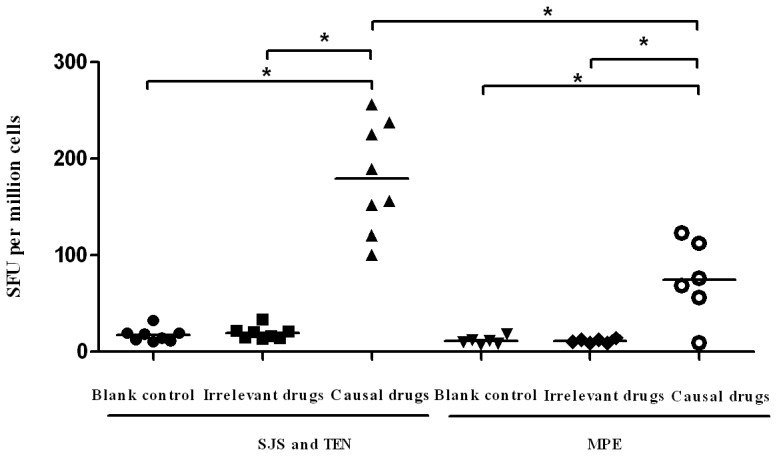
*Ex vivo* IFN-γELISPOT responses to causal drugs. Purified PBMC (1×10^5^ in 100 μL ) from patients with SJS and TEN or MPE were stimulated for 24 h with causal drugs, irrelevant drugs or medium alone in 96-well ELISpot plate. Responses are presented as number of IFN-γ-positive spot-forming units (SFU) per million cells. Each data point represents an individual patient with SJS and TEN or MPE. Median is indicated by a horizontal line. *P<0.01.

Next, we investigated IFN-γ effector response by using an *ex vivo* overnight IFN-γ ELISpot assay. We observed significant IFN-γ responses against causal drugs by PBMCs from all the 8 patients. The response ranged from 100 to 237 SFU per million cells (Fig. 2). These responses were drug-specific as stimulation with chemically irrelevant drugs had no IFN-γ production. Again, we did not observe a significant correlation between *ex vivo* IFN-γ ELISpot response and the time interval from previous SJS and TEN. In patient 1, the response reached 152 SFU per million PBMCs, which was significantly higher than the positive cutoff. Five of six patients with previous MPE also had significant *ex vivo* IFN-γ ELISpot responses (Fig. 2). The positive responses ranged from 56 to 123 SFU per million PBMCs, which were significant lower than those in SJS and TEN group. These data show that drug-specific T cells in SJS and TEN patients after remission are detectable *ex vivo* and show rapid effector function to causal drugs.

### sFasL production by drug-specific effector memory T cells

**Figure 3:**
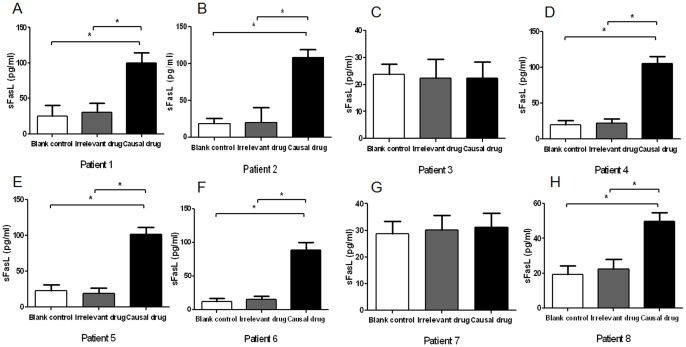
sFasL levels in supernatants of drug-stimulated PBMCs from SJS and TEN patients. PBMCs from patients with SJS and TEN were cultured with causal drugs, irrelevant drugs, or medium alone for 3 days. The supernatants were collected and the concentrations of sFasL were determined by sFasL ELISA kit. A–H, Patient 1–8. The graphs show the means of three samples of each patient and error bars represent SD values. *P<0.05.

**Figure 4:**
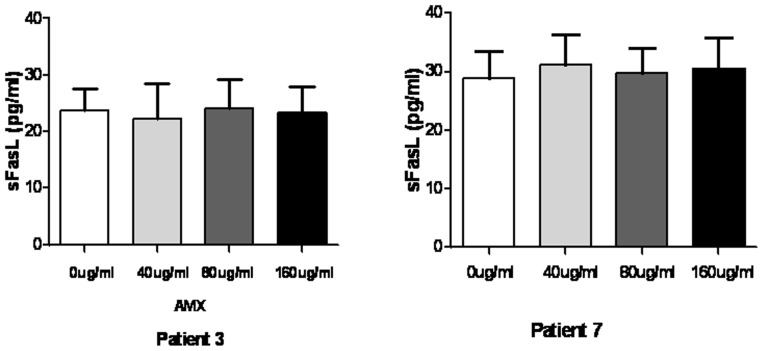
No change of sFasL level in supernatants of PBMCs upon stimulation with higher concentration of amoxicillin. PBMCs from patient 3 and patient 7 were cultured with amoxicillin at concentrations of 0, 40, 80,160 μg/ml. The sFasl levels were then determined by ELISA. A. Patient 3. B, Paitent 7. Results represent mean ± SD from three independent experiments. *P<0.05.

**Figure 5:**
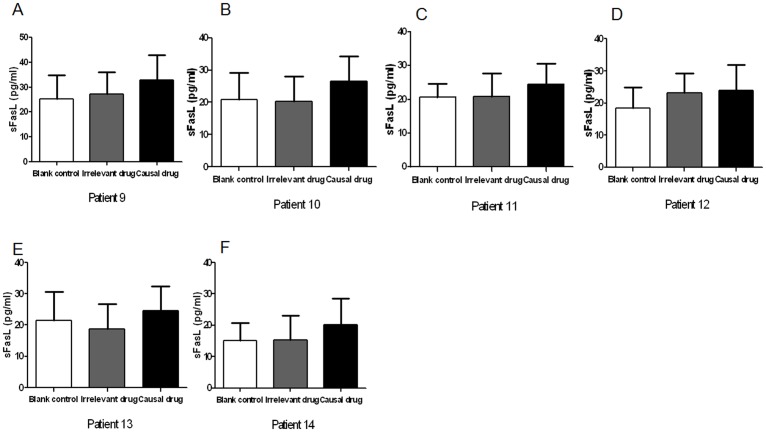
sFasL levels in supernatant of drug-stimulated PBMCs from MPE patients. PBMCs from patients with MPE were cultured with causal drugs, irrelevant drugs, or medium alone for 3 days. The supernatants were collected and the concentrations of sFasL were determined by sFasL ELISA kit. A–F, Patient 9–14. The graphs show the means of three samples of each patient and error bars represent SD values.

We proceed to investigate sFasL effector memory response in SJS and TEN patients after remission. PBMCs freshly isolated from SJS and TEN patients were cultured with different causal drugs for 3 days and the culture supernatants were examined for sFasL. In 6 of 8 patients (Patient 1, 2, 4, 5, 6, 8), significant increased sFasL productions by PBMC were observed, whereas no response was seen in the other 2 patients (Patient 3, 7) (Fig. 3). The increased sFasL response was drug-specific as well, as no significant increase of sFasL was found in the samples upon stimulations with chemically irrelevant drugs. In the 2 patients yielding negative sFasL response, the causal drug was amoxicillin and the time interval was 1 year and 2 months respectively. To eliminate the possibility that the absence of sFasL response in the 2 patients might be due to insufficient concentration of causal drug stimulation, we further used a series of higher concentrations of amoxicillin to stimulate the PBMCs. We could not detect any changes of sFasL level in the supernatant of PBMCs upon stimulation with higher concentration of amoxicillin (Fig. 4). In patients with previous MPE, PBMCs showed higher levels of sFasL secretion by stimulating with the causal drugs. However, there was no significant difference between the levels of sFasL in blank control, chemically irrelevant drugs-stimulated and causal drugs-stimulated groups (Fig. 5).

**Figure 6:**
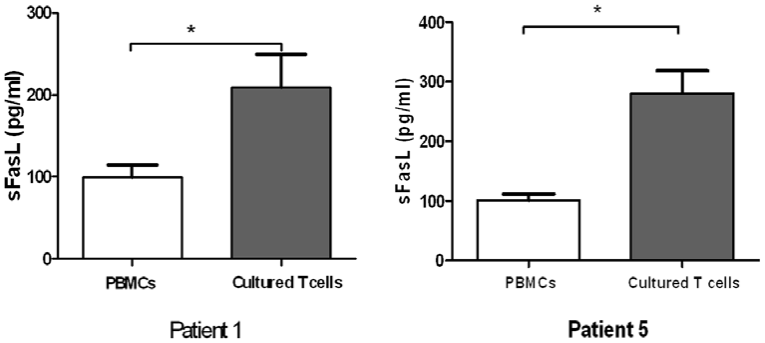
Comparison of sFasL release between PBMCs and cultured T cells. PBMCs from patient 1 and 5 were cultured with causal drugs for 10 days. The cells were subsequently washed, and re-stimulated with causal drugs for 3 days. The sFasL levels in supernatants were determined by sFasL ELISA kit and were compared with those in 3-day PBMC culture supernatants. Results represent mean ± SD from three independent experiments. *P<0.05.

To further investigate sFasL-mediated memory response, we harvested the *in vitro* cultured T cells from Patient 1 and 5, and re-stimulated the T cells with the relevant drugs for 3 days. Compared with freshly isolated PBMCs, the cultured T cells released higher levels of sFasL on stimulation with the causal drugs (2.0-folds and 2.8 folds, respectively). This increase was also drug specific because treatment with the chemically irrelevant drug had no effect (Fig. 6). These findings were consistent with IFN-γ memory in the patients, suggesting that the sFasL-mediated memory responses are also maintained in SJS and TEN patients after remission.

### sFasL-mediated toxicities of PBMC supernatant against keratinocytes

**Figure 7:**
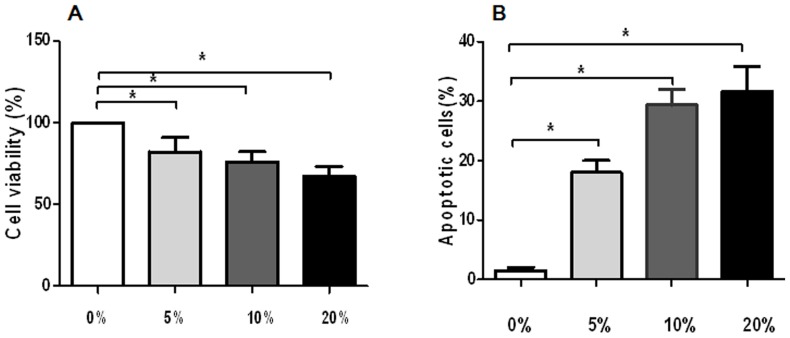
*In vitro* toxicities of PBMC supernatant against keratinocytes. HaCat cells were incubated with culture medium alone or culture medium containing 5%, 10%, 20% PBMC supernatants for 24 hours. A. MTT assays showed that the supernatants of drug-stimulated PBMCs were cytotoxic against keratinocytes in a dose-dependent manner. B. Annexin V staining showed that the supernatants of drug-stimulated PBMCs induced apoptosis of keratinocytes in a dose – dependent manner. *P<0.05.

**Figure 8:**
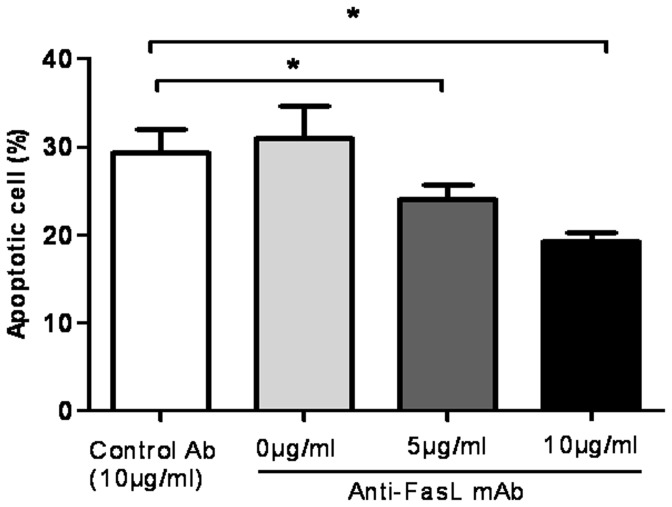
sFasL mAb blocking decreased the percentage of apoptotic keratinocytes. HaCat cells were incubated with culture medium containing 10% PBMC supernatants, the neutralizing anti-FasL mAb (NOK-2), the negative control mAb for 24 hours. Annexin V staining showed that the inhibitory anti-FasL mAb reduced the number of apoptotic cells in a dose-dependent manner. *P<0.05.

We next examine sFasL-mediated toxicity of PBMC supernatant against keratinocytes. HaCat cells, a keratinocyte cell line, were incubated with culture medium alone, or culture medium containing 5%, 10%, 20% PBMC supernatants from the 6 patients (Patient 1, 2, 4, 5, 6, and 8), the neutralizing anti-FasL mAb, the negative control mAb. Cell viability was measured by MTT assays. We observed that the supernatant of drug-stimulated PBMCs were cytotoxic against keratinocytes (Fig. 7). To investigate the mechanism of its cytotoxicity, HaCat cells were further stained with Annexin-V and analyzed by flow cytometry. The addition of drug-stimulated PBMC supernatant to the medium induced keratinocyte apoptosis, whereas keratinocytes incubated with medium alone showed significantly less apoptotic cell death (Fig. 7). Moreover, culture with an inhibitory anti-FasL mAb reduced the percentages of apoptotic cells in a dose-dependent manner, whereas the negative control mAb failed to mediate apoptosis (Fig. 8). These data suggest that T cell supernatant mediates keratinocyte apoptotic death involving Fas and sFasL.

## Discussion

The long persistence of memory has been observed previously *in vivo* because some patients respond to minute amounts of the drug with severe reactions even years after the initial hypersensitivity reaction [17]. However, there are only a few studies that have attempted to examine memory responses over time. The cultured ELISpot assay was originally developed to identify protective T-cell responses against malaria infection [14], [18]. This method not only magnifies the response by allowing the T cells present to divide, but also enables the resting central memory T cells to differentiate into effector cells. Specifically, the cultured IFN-γ ELISpot has been used to identify long-lasting memory T-cell responses. In the present study, we have now examined the durability of memory T cell response in SJS and TEN patients after remission by using the cultured IFN-γ ELISpot assay. In all 8 patients, we observed substantial IFN-γresponse in the restimulation phase of the assay. The response appear to be strong as about 2565 to 4400 IFN-γ producing cells were observed per million T cells. Thus, we conclude that T cell memory responses are stably maintained in SJS and TEN patients even after long-term remission.

Using *ex vivo* ELISpot assay, we also detected significant IFN-γ production after overnight stimulation with causal drugs. The frequency we detected varied from 100 to 256 IFN-γ producing cells per million PBMCs, which is comparable with previous reports that show about 1∶250 to 1∶10,000 of drug-reactive T cells exist in patients with severe drug allergy [12]. Of the 8 patients we enrolled, we did not observe a decline of IFN-γ response with the increased time intervals. These data show that IFN-γ effector function of memory T cells is also stably maintained in SJS and TEN patients in remission.

Our results further emphasize that IFN-γ release assay may serve as an *in vitro* diagnostic test for SJS and TEN. Indeed, previous studies demonstrated that increased IFN-γ release could be observed in patients with a wide variety of CADRs after *in vitro* challenge of the patients' lymphocytes with the culprit drugs [19], [20], [21]. In another study, it was found that IFN-γ tests were positive in 77.8% of the patients with a variety of CADMS for 49.0% of the drugs. In addition, the proportion of positive IFN-γ tests was directly associated with the degree of drug suspicion. More importantly, the high proportion (100%) of *in vitro* IFN-γ release was seen in patients with drug-induced bullous eruptions [22]. In our study, all the SJS and TEN patients exhibited circulating IFN-γ-producing T cells upon *ex vivo* restimulation with the drugs, which, in line with previous studies [20], [22], suggests that *in vitro* IFN-γ release tests could be useful for identifying the drugs associated with SJS and TEN. Furthermore, because the present study was performed over a long period after the occurence of SJS and TEN, it indicates that this assay may be helpful in predicting the potential risk of re-exposure of the drugs to cause SJS and TEN. Of note, the present study comprised only limited number of SJS and TEN patients, and the number of SJS patients was less than TEN patients we enrolled. Future studies will be needed to provide data on the sensitivity and the specificity of this assay in a larger number of SJS and TEN patients and controls.

Significant evidence exists implicating sFasL-mediated toxicities in the occurrence of SJS and TEN. In patients with SJS and TEN, sFasL level in serum is increased significantly before or after onset of skin damage [23], [24], [25]. PBMCs from patients with SJS and TEN secrete significant amounts of sFasL in the acute stage after stimulation with causal drugs, which subsequently induce keratinocyte apoptosis in a dose-dependent manner [8]. Moreover, ibuprofen-induced SJS or sulfonamides-induced hypersensitivity syndrome had significantly elevated levels Fas upon drug re-stimulations 2–4 years after disease remission [10], [11].

Considering the potential role of sFasL in kerationcytes apoptosis, we examined the sFasL production by drug-stimulated PBMCs as an indication of effector memory response. Strikingly, we observed significant sFasL productions from PBMCs in 6 of 8 patients with SJS and TEN. The sFasL levels are significantly higher than those with previous MPE. The sFasL response is drug-specific as no significant increase of sFasL was found in the samples upon stimulation with chemically irrelevant drugs. Furthermore, the T cells after 10-day culture released more sFasL than the freshly isolated PBMCs on re-stimulation with the causal drugs, indicating sFasl-producing cells could be expanded *in vitro* in the presence of the causal drugs. Additionally, our study showed that sFasL produced by PBMCs of SJS and TEN patients exert an anti-proliferative and apoptotic effects against keratinocytes. Collectively, these findings, in line with previous reports [8], [10], [11], confirming that the sFasL-mediated toxicities could be sustained in patients with SJS and TEN after remission.

Interestingly, no significant sFasL production was observed in 2 of 8 patients. The negative sFasL production was further proved even if we used higher concentrations of causal drugs to simulate PBMCs. We considered that the results should not be interpreted too quickly as suggesting loss of sFasL response in some patients with SJS and TEN after remission. In Murata' study, he detected increased sFasL levels in 5 of 7 patients with SJS/TEN before onset. There were 2 patients who did not show a signifncant increase of sFasL level in acute stage. Therefore, it is possible that our inability to demonstrate sFasL response in these 2 cases may be due to the lack of sFasL response in the acute stage. Further studies are necessary to clarify this.

At present it is still difficult to explain the mechanism accounting for persistent, drug-specific memory in patient with SJS and TEN. It has been suggested that persisting antigen might help sustenance of effector and memory T cells [26], [27]. In our study, because the symptoms had been severe in all cases, all patients had strictly avoided the incriminated drugs in the whole period after remission. In addition, no chemically related drugs were used by these patients to avoid potential cross-reactivity. This indicates that T cells may not require antigen re-exposure for their maintenance. Further studies are needed to address the mechanism of persistent drug-reactivity and effector function of T cells in patients with SJS and TEN.

In summary, we demonstrate that the IFN-γ and sFasL memory could be maintained, and recalled over an extended period of at least 3 years. The data contribute to our understanding of the development and maintenance of drug-specific memory with consequent potential clinical implications in identifying causal drugs in SJS and TEN patients. We think that IFN-γ and sFasL-based assay might be promising tools in SJS and TEN diagnosis.

## Materials and Methods

### Ethics Statement

The study was approved by Ethics Committee of the Xijing Hospital, Fourth Military Medical University. All participants gave written informed consent.

### Patients and clinical samples

Eight patients with a very well characterized medical history of SJS and TEN and eight patients with a history of drug-induced maculopapular exanthema (MPE) were enrolled. A summary of the patient characteristics is given in Table 1. PBMCs were separated from heparinized peripheral blood using Lymphoprep (1114547, Nycomed, Oslo, Norway) density gradient. PBMCs were then washed in RPMI supplemented with and L-glutamine (R0) and resuspended in RPMI with L-glutamine, and 10% FCS (R10).

### Drugs

Nontoxic concentrations of the drugs were used for *in vitro* stimulations. The following drugs were used: amoxicillin, nimesulide, acetaminophen, hydroxychloroquine, allopurinol, carbamazepine (all from Sigma-Aldrich, St Louis, MO, USA). Solution form infusion was used for penicillin and cephazolin (Harbin Pharma Co, Harbin, China).

### T cell culture

Fresh PBMCs at 2×10^6^/mL in R10 were incubated on 24-well flat bottom culture plate with causal drugs at different concentrations for 10 days at 37°C 5% CO_2_. On days 3, 6 and 9, the cells were supplemented with R10 plus 200 IU/mL human IL-2. On day 10, cells were removed from the plate, washed twice in sterile PBS, and returned to a clean well overnight in R10 to rest before ELISpot analysis on day 11.

### IFN-γ ELISpot

Ninety-six–well multiscreen Immobilon-P plates (IP-plates) (MAIPS4510, Millipore, Watford, UK) were coated with 50 uL per well at 15 mg/mL with IFN-γ catcher antibody (3420-2A, Mabtech, Nacka Strand, Sweden) in sterile PBS, pH 7.4, and incubated 16 hours at 4°C. Plates were washed 6 times with R0 and then blocked with R10 for 1 hour at 37°C. Cells were added to IFN-γ mAb-coated plates at 1×10^6^/well (PBMCs), or 2×10^5^/well (cultured T cells). Then, the causal drugs were added to the wells to the final concentrations shown in table 1. The cells were incubated with the drugs for 24 hr at 37°C and 5% CO_2_. Phytohemagglutinin was used as the positive control at 2 mg/mL, and R0 as a blank control. For each individual, a chemically irrelevant drug was used to assess nonspecific IFN-γ responses (Table 1). The plates were then developed by the addition of 1 μg/ml of biotin-linked anti-IFN-γ MAb (MAb 7-B6-1-biotin; Mabtech) as a detection antibody, which was subsequently conjugated to streptavidin alkaline phosphatase (Mabtech) and visualized using an AP conjugate substrate kit (Bio-Rad). Spots were enumerated using an automated AID ELISpot reader. The background (cells only) was subtracted, and the data were expressed as the number of spot-forming units (SFU) per 10^6^ cells.

### Enzyme-Linked Immunosorbent Assay (ELISA)

Fresh PBMC (2×10^6^/ml) were cultured with R10 alone, R10 with chemically irrelevant drugs, or R10 with causal drugs for 3 days at 37°C and 5% CO_2_. For patients 1 and 5, the T cells after 10-day *in vitro* culture were harvested and re-stimulated with causal drugs for 3 days. The supernatants were collected and the concentrations of sFasL were determined by sFasL ELISA kit (MBL, Nagoya, Japan). The developed reaction was quantified by reading at 490 nm. Each individual sample was analyzed in triplicate.

### In vitro cytotoxicity

Human keratinocytes (HaCaT) were plated into culture plates at a density of 1×10^6^ cells/ml, and cultured until just before confluence. Then, the cells were incubated at 37°C for 24 hours with R10 containing drug-stimulated PBMC supernatant (5%, 10%, 20%) or drug-stimulated PBMC supernatant plus an inhibitory anti-FasL mAb (NOK-2, PharMingen, San Diego, CA) or the negative control mAb. Cell viability was assessed with the MTT assay. Detached cells and trypsinized adherent cells were also collected. Cells were stained with annexin-V and analyzed on a BD Phar-Mingen cell sorter.

### Statistical Analysis

Statistical analyses were calculated by using Prism software (GraphPad, San Diego, CA). A Wilcoxon signed rank test was used for comparison of paired conditions. A Mann-Whitney test was used for unpaired data. A P-value less than 0.05 was considered to be significant.

## References

[1] Pichler WJ, Naisbitt DJ, Park BK (2011) Immune pathomechanism of drug hypersensitivity reactions. J Allergy Clin Immunol 127: S74–81. 21354503 10.1016/j.jaci.2010.11.048

[2] Gerber BO, Pichler WJ (2004) Cellular mechanisms of T cell mediated drug hypersensitivity. Curr Opin Immunol 16: 732–737. 15511665 10.1016/j.coi.2004.09.016

[3] Chung WH, Hung SI, Yang JY, Su SC, Huang SP, et al (2008) Granulysin is a key mediator for disseminated keratinocyte death in Stevens–Johnson syndrome and toxic epidermal necrolysis. Nat Med 14: 1343–1350. 19029983 10.1038/nm.1884

[4] Nassif A, Bensussan A, Dorothée G, Mami-Chouaib F, Bachot N, et al (2002) Drug specific cytotoxic T-cells in the skin lesions of a patient with toxic epidermal necrolysis. J Invest Dermatol 118: 728–733. 11918724 10.1046/j.1523-1747.2002.01622.x

[5] Nassif A, Bensussan A, Boumsell L, Deniaud A, Moslehi H, et al (2004) Toxic epidermal necrolysis: effector cells are drug-specific cytotoxic T cells. J Allergy Clin Immunol 114: 1209–1215. 15536433 10.1016/j.jaci.2004.07.047

[6] Posadas SJ, Padial A, Torres MJ, Mayorga C, Leyva L, et al (2002) Delayed reactions to drugs show levels of perforin, granzyme B, and Fas-L to be related to disease severity. J Allergy Clin Immunol 109: 155–161. 11799383 10.1067/mai.2002.120563

[7] Abe R (2008) Toxic epidermal necrolysis and Stevens–Johnson syndrome: soluble Fas ligand involvement in the pathomechanisms of these diseases. J Dermatol Sci 52: 151–159. 18657400 10.1016/j.jdermsci.2008.06.003

[8] Abe R, Shimizu T, Shibaki A, Nakamura H, Watanabe H, et al (2003) Toxic epidermal necrolysis and Stevens–Johnson syndrome are induced by soluble Fas ligand. Am J Pathol 162: 1515–1520. 12707034 10.1016/S0002-9440(10)64284-8 PMC1851208

[9] Arnold R, Seifert M, Asadullah K, Volk HD (1999) Crosstalk between keratinocytes and T lymphocytes via Fas/Fas ligand interaction: modulation by cytokines. J Immunol 162: 7140–7147. 10358159

[10] Neuman MG, Shear NH, Malkiewicz IM, Taeri M, Shapiro LE, et al (2007) Immunopathogenesis of hypersensitivity syndrome reactions to sulfonamides. Transl Res 149: 243–253. 17466923 10.1016/j.trsl.2006.12.001

[11] Neuman M, Nicar M (2007) Apoptosis in ibuprofen-induced Stevens–Johnson syndrome. Transl Res 149: 254–259. 17466924 10.1016/j.trsl.2006.12.005

[12] Beeler A, Engler O, Gerber BO, Pichler WJ (2006) Long-lasting reactivity and high frequency of drug-specific T cells after severe systemic drug hypersensitivity reactions. J Allergy Clin Immunol 117: 455–462. 16461148 10.1016/j.jaci.2005.10.030

[13] Zawodniak A, Lochmatter P, Yerly D, Kawabata T, Lerch M, et al (2010) In vitro detection of cytotoxic T and NK cells in peripheral blood of patients with various drug-induced skin diseases. Allergy 65: 376–384. 19793058 10.1111/j.1398-9995.2009.02180.x

[14] Reece WH, Pinder M, Gothard PK, Milligan P, Bojang K, et al (2004) A CD4(+) T-cell immune response to a conserved epitope in the circumsporozoite protein correlates with protection from natural Plasmodium falciparum infection and disease. Nat Med 10: 406–410. 15034567 10.1038/nm1009

[15] Lalvani A, Brookes R, Hambleton S, Britton WJ, Hill AV, et al (1997) Rapid effector function in CD8+ memory T cells. J Exp Med 186: 859–865. 9294140 10.1084/jem.186.6.859 PMC2199056

[16] Godkin AJ, Thomas HC, Openshaw PJ (2002) Evolution of epitope-specific memory CD4(+) T cells after clearance of hepatitis C virus. J Immunol 169: 2210–2214. 12165552 10.4049/jimmunol.169.4.2210

[17] Schnyder B, Helbling A, Kappeler A, Pichler WJ (1998) Drug-induced papulovesicular exanthema. Allergy 53: 817–818. 9722238 10.1111/j.1398-9995.1998.tb03985.x

[18] Keating SM, Bejon P, Berthoud T, Vuola JM, Todryk S, et al (2005) Durable human memory T cells quantifiable by cultured enzyme-linked immunospot assays are induced by heterologous prime boost immunization and correlate with protection against malaria. J Immunol 175: 5675–5680. 16237057 10.4049/jimmunol.175.9.5675

[19] Halevy S, Cohen A, Livni E (2000) Acute generalized exanthematous pustulosis associated with polysensitivity to paracetamol and bromhexine: the diagnostic role of in vitro interferon-gamma release test. Clin Exp Dermatol 25: 652–654. 11167983 10.1046/j.1365-2230.2000.00729.x

[20] Halevy S, Cohen AD, Livni E (1999) The diagnostic role of the in vitro drug-induced interferon-gamma release test in Stevens–Johnson syndrome. Int J Dermatol 38: 835–840. 10583616 10.1046/j.1365-4362.1999.00792.x

[21] Halevy S, Gold I, Cohen AD, Grossman N (2004) In vitro interferon-gamma release test in the diagnosis of drug-induced erythema nodosum. Isr Med Assoc J 6: 59–60. 14740516

[22] Halevy S, Cohen AD, Grossman N (2005) Clinical implications of in vitro drug-induced interferon gamma release from peripheral blood lymphocytes in cutaneous adverse drug reactions. J Am Acad Dermatol 52: 254–261. 15692470 10.1016/j.jaad.2004.05.006

[23] Viard I, Wehrli P, Bullani R, Schneider P, Holler N, et al (1998) Inhibition of toxic epidermal necrolysis by blockade of CD95 with human intra-venous immunoglobulin. Science 282: 490–493. 9774279 10.1126/science.282.5388.490

[24] Chang HY, Cooper ZA, Swetter SM, Marinkovich MP (2004) Kinetics and specificity of fas ligand induction in toxic epidermal necrolysis. Arch Dermatol 140: 242–244. 14967808 10.1001/archderm.140.2.242

[25] Murata J, Abe R, Shimizu H (2008) Increased soluble Fas ligand levels in patients with Stevens–Johnson syndrome and toxic epidermal necrolysis preceding skin detachment. J Allergy Clin Immunol 122: 992–1000. 18692887 10.1016/j.jaci.2008.06.013

[26] Shin H, Blackburn SD, Blattman JN, Wherry EJ (2007) Viral antigen and extensive division maintain virus-specific CD8 T cells during chronic infection. J Exp Med 204: 941–949. 17420267 10.1084/jem.20061937 PMC2118542

[27] Cush SS, Anderson KM, Ravneberg DH, Weslow-Schmidt JL, Flaño E (2007) Memory generation and maintenance of CD8+ T cell function during viral persistence. J Immunol 179: 141–153. 17579032 10.4049/jimmunol.179.1.141 PMC3110076

